# Exploratory EEG correlates of sensory and affective pain dimensions in patients with chronic widespread pain

**DOI:** 10.3389/fpain.2026.1705011

**Published:** 2026-07-16

**Authors:** Keita Ueno, Keiko Yamada, Masaya Ueda, Yasuo Naito, Ryouhei Ishii

**Affiliations:** 1 Graduate School of Rehabilitation Science, Osaka Metropolitan University, Osaka, Japan; 2 Department of Occupational Therapy, Morinomiya University of Medical Sciences, Osaka, Japan; 3 Pain Medicine, Juntendo University Graduate School of Medicine, Tokyo, Japan; 4 Department of Anesthesiology and Pain Medicine, Juntendo University Faculty of Medicine, Tokyo, Japan; 5 Department of Psychiatry, Osaka University Graduate School of Medicine, Osaka, Japan

**Keywords:** chronic pain, chronic widespread pain, electroencephalography, exact low-resolution electromagnetic tomography, functional connectivity

## Abstract

**Introduction:**

Chronic widespread pain (CWP) is a debilitating condition characterized by persistent pain, yet objective biomarkers based on electroencephalography (EEG) remain elusive. This exploratory study aimed to identify neurophysiological correlations related to the sensory and affective dimensions of CWP using resting-state EEG data analyzed with exact low-resolution electromagnetic tomography (eLORETA).

**Methods:**

We analyzed data from 20 CWP patients and 22 healthy controls, calculating current source density (CSD) and functional connectivity (FC) in key brain networks and correlating them with pain scores from the numerical rating scale and short-form McGill Pain Questionnaire.

**Results:**

While no significant CSD or FC differences emerged between the groups, analysis within the CWP group yielded two preliminary associations. First, current pain intensity was negatively correlated with CSD values in the γ bands of the precuneus/posterior cingulate cortex (PCC) (r = −0.743). Second, the affective pain component was positively correlated with δ band FC between the anterior cingulate cortex (ACC) and the right anterior insula (r = 0.647).

**Discussion:**

These results suggest that EEG-derived neurophysiological correlations can offer insights into individual differences in the CWP experience. The findings in the precuneus/PCC may reflect exploratory correlations associated with sensory pain intensity, while the salience network connectivity may be linked to the affective dimension of pain. Future research with larger, homogeneous samples and longitudinal designs is needed to evaluate the potential clinical relevance of these electrophysiological correlations.

## 1 Introduction

Chronic widespread pain (CWP) is characterized by persistent pain across multiple body regions and is a key symptom in conditions such as fibromyalgia, where no clear tissue damage or lesions are present (1). CWP prevalence ranges from 1.4% to 24.0%, influenced by psychosocial factors (2). In 2017, the International Association for the Study of Pain introduced the concept of “nociplastic pain,” defined as pain arising from altered nociception without evidence of tissue damage or somatosensory system lesions. Furthermore, pain is increasingly recognized as a multidimensional experience that involves not only sensory perception but also complex emotional and suffering components (3). This concept is critical for understanding of CWP, but reliable neurophysiological indicators remain elusive.

Functional magnetic resonance imaging (fMRI) studies have revealed altered functional connectivity (FC) in patients with CWP, including decreased FC in the default mode network (DMN) and increased FC in the salience network (SN) (4). In fibromyalgia, increased connectivity between the DMN and insular cortex, a key pain-processing region (5) (4), has been observed, along with a correlation between reduced connectivity and clinical pain relief (6) (5). Additionally, patients with fibromyalgia show heightened neuronal activation in pain-processing brain regions under pressure stimulation compared with healthy controls (HCs) (7) (6). Notably, previous research has suggested that distinct brain systems mediate the effects of nociceptive input and affective self-regulation on pain (8). These findings suggest that brain network modulation and activity changes in pain-processing regions may provide objective insights into the neurophysiological basis of clinical pain. However, frequent fMRI scans are impractical because of patient burden, and potential distress (9).

In recent years, electroencephalography (EEG) studies of chronic pain have become increasingly popular. EEG is non-invasive, relatively inexpensive, and has high temporal resolution, making it easy to perform time-series evaluations and highly applicable to clinical practice. Characteristic EEG findings in patients with chronic pain include increased EEG power and slowed dominant frequency (10), increased *θ* and *α* wave power (11), and increased connectivity in the frontal *θ* and *γ* bands (12). We discovered changes in brain networks and current source density (CSD) on the basis of electrical activity in individuals with chronic low back pain using eLORETA (exact low-resolution electromagnetic tomography) (13). However, specific EEG components characteristic of CWP have not been identified, and findings remain inconsistent across studies, likely due to the high clinical heterogeneity of the condition. Therefore, investigating EEG correlates associated with CWP is a valuable step. Owing to the current lack of consensus in the literature regarding consistent EEG biomarkers for CWP, we conducted an exploratory investigation rather than testing a narrow, predefined hypothesis. Following previous research, we aim to examine the network modulation and local brain activity changes using eLORETA, and to explore the modulation of brain networks and CSD. We compare the CSD and FC of the DMN and SN in the resting-state EEGs of patients with CWP and healthy subjects, and exploratively investigate their relationships with both the sensory and affective dimensions of pain, as reflected in various clinical assessment scores. This study has the potential to provide exploratory insights into the neurophysiological correlations of CWP and inform future research on EEG-based clinical assessments.

## 2 Material and methods

### 2.1 Participants

Patient and public involvement was not undertaken in any aspect of this study, including the design, conduct, reporting, or dissemination of our research.

This study extracted and analyzed a specific subset of data from a larger open EEG dataset collected at the Technical University of Munich (14) (12). To ensure a homogeneous study population, we selected all available participants from the sub-study focused on Fibromyalgia. The final sample consisted of 20 patients who met the clinical diagnosis of CWP and 22 healthy controls (HCs) from the same recording cohort. All participants gave their written informed consent prior to participation in the study.

Participants in the CWP group were required to have experienced pain for at least 6 months, with an average pain intensity reported to be 4 or higher on a scale from 0 (no pain) to 10 (worst pain imaginable) over the past 4 weeks. The exclusion criteria were rapid changes in pain status (e.g., in relation to trauma or surgery) within the past 3 months; major neurological disorders such as stroke, epilepsy, or dementia; serious psychiatric conditions other than depression; and severe systemic diseases. In addition, because benzodiazepine medications have been reported to have a significant effect on EEG (15), patients taking these medications were also excluded. Other medications were not restricted. The exclusion criteria for the HC group were a history of pain lasting more than 6 months; experience with examinations, surgery, or acute trauma in the past 3 months; pain on the day of the examination; and neurological or psychiatric disorders.

Immediately before the EEG measurement, participants filled out questionnaires related to pain characteristics and any comorbid conditions. Current pain intensity ratings were obtained from the short-form McGill Pain Questionnaire (SF-MPQ), which uses a visual analogue scale anchored at 0 (no pain) and 100 (worst imaginable pain). These ratings were divided by 10. Pain characteristics were assessed using the SF-MPQ which provides sensory (0–33) and affective (0–12) subscores, as well as a total score (0–45) (16) (14). Although these two dimensions are often interrelated, the SF-MPQ is a validated instrument designed to dissociate these aspects. In this study, we evaluated the correlation between these scores to confirm their relationship within our sample before exploring their distinct neurophysiological correlations. Furthermore, depression was assessed using the Beck Depression Inventory–Second Edition (BDI-II; total score range 0–63), and anxiety was assessed using the State-Trait Anxiety Inventory (STAI). For the STAI, the total score (range 40–160) was utilized for analysis without differentiating between the state and trait anxiety subscales.

### 2.2 EEG recording

EEG recordings were obtained with 64 electrodes and a BrainAmp MR plus amplifier system (Brain Products, Munich, Germany). The electrodes were placed according to the international 10–20 system, with 19 channels plus Fpz, CPz, POz, Oz, Iz, AF3/4, F5/6, FC1/2/3/4/5/6, FT7/8/9/10, C1/2/5/6, and CP1/2/3/4. Two electrodes were placed at the outer canthi of both eyes to monitor eye movements. The reference electrode was FCz, and the ground electrode was AFz. The sampling frequency was 1,000 Hz, and a bandpass filter was applied in the range of 0.016–250 Hz. Impedance during measurement was kept below 20 kΩ.

Participants were assessed for 5 min each in an eyes-open state and an eyes-closed state while remaining awake, relaxed, and at rest. In this study, we analyzed the eyes-closed data, and we extracted and analyzed 19 channels on the basis of the international 10–20 system, as alluded to previously. While the original data were recorded with 64 channels, we focused on the 19-channel configuration to ensure clinical translatability and feasibility, as this is the global standard in routine clinical EEG. Although increased sensor density generally improves source localization accuracy (17), a 19-channel setup has been shown to be reliable and sufficient for characterizing the global properties of brain network modulations in eyes-closed resting-state conditions (17, 18).

### 2.3 Preprocessing

The EEGLAB toolbox (19) (15), which runs in the MATLAB (MathWorks, Natick, MA) environment, was used for EEG data preprocessing. EEGLAB, an open-source toolbox for the analysis of single-trial EEG dynamics, includes functions such as independent component (IC) analysis. The data were resampled from 1,000 Hz to 500 Hz, followed by the application of a bandpass filter between 1 and 50 Hz. Furthermore, 50 Hz of power line noise was removed using the CleanLine plugin of EEGLAB, a method recommended by Mitra and Bokil (20).

Next, artifact subspace reconstruction was performed to correct burst artifacts. The subspace reconstruction algorithm detects data segments that exceed a predefined standard deviation threshold and reconstructs them by projecting them into a subspace that reflects clean signals. In this study, the maximum allowable standard deviation was set to 20 for a 0.5-second window. This technique reduces the impact of artifacts while preserving the essential brain activity information. After that, IC analysis was performed using the RunICA function, and an artifact identification value was calculated for each IC using ICLabel (21). ICs with a brain identification value of less than 90% and identification values of 90% or more for other labels were removed, and the EEG waveform was reconstructed.

### 2.4 EEG data analysis

#### 2.4.1 Current source density (CSD)

The distribution of cortical CSD was analyzed using eLORETA (22) (18). eLORETA is a linear weighted minimum norm-based inverse solution that can reconstruct cortical electrical activity from scalp EEG data. It not only estimates CSD localization but also evaluates FC between brain regions while minimizing the effects of volume conduction and low spatial resolution. The eLORETA head model and electrode coordinates are derived from the Montreal Neurological Institute's 152 average MRI brain map, divided into 6,239 voxels (each voxel has a spatial resolution of 5 mm) and limited to the cortical gray matter. The validity of eLORETA has been verified in past studies using fMRI (23, 24), structural MRI (25), positron emission tomography (26), and intracranial EEG (27).

Following preprocessing, artifact-free EEG data were segmented into 2-second epochs for eLORETA analysis. The frequency bands analyzed were as follows:

*δ* (1.5–4.0 Hz)

*θ* (4.5–7.5 Hz)

*α*1 (8.0–10.0 Hz)

*α*2 (10.5–13.0 Hz)

*β*1 (13.5–20.0 Hz)

*β*2 (20.5–30.0 Hz)

*γ* (30.5–50.0 Hz)

These frequency boundaries were selected based on established eLORETA protocols (28) to capture distinct neurophysiological processes and maintain methodological consistency with previous resting-state research.

Using eLORETA, CSD values in each of the seven frequency bands were estimated for each of the 6,239 voxels. Thereafter, group differences in CSD values in each frequency band were evaluated using an unpaired t-test on a voxel-by-voxel basis.

In addition, correlation analysis was performed in the CWP group only, between CSD values and self-reported scores related to pain symptoms (current pain intensity, SF-MPQ [sensory representation items, affective representation items, total score], Beck Depression Inventory–Second Edition, and State-Trait Anxiety Inventory).

#### 2.4.2 Functional connectivity (FC)

In the FC analysis, the DMN (medial prefrontal gyrus, posterior cingulate cortex [PCC], left and right inferior parietal lobules) (29, 30) and the SN (left anterior insular cortex and right anterior insular cortex [rAI], anterior cingulate cortex [ACC]) (31) were set as regions of interest. Specifically, we employed the “all nearest voxels” method in eLORETA, in which each of the 6,239 cortical voxels is assigned to its closest predefined seed. This approach partitions the entire cortex into distinct ROIs, allowing for a comprehensive assessment of network-level interactions rather than point-to-point connections. This choice is supported by fMRI evidence showing that chronic pain conditions involve a functional reorganization of the DMN (32), a finding further supported by systematic evidence of consistent functional and structural DMN abnormalities in chronic pain populations (33). Furthermore, in patients with CWP, connectivity within the DMN and SN has been shown to be disrupted and specifically modulated by pain sensitivity (4). This focus on the SN is also justified by its recognized role in integrating salient sensory information with the cognitive and affective processing deficits commonly observed in clinical pain states (34). Our analysis aimed to bridge these fMRI findings with electrophysiological data to clarify the spectral characteristics of these network alterations. The preprocessed EEG data were divided into epochs in the same way as in the CSD analysis, and lagged linear connectivity (35) was calculated in each frequency band (hereinafter referred to as the FC value). Lagged linear connectivity is an index that assumes that the activity in one region affects the activity in another region, with a time delay, and eliminates spurious correlations caused by volume conduction by eliminating zero-delay immediate interactions. Group differences in FC between the regions of interest were evaluated with an unpaired t-test using eLORETA. As with CSD, a correlation analysis was also conducted between FC and self-reported scores.

### 2.5 Multiple comparison correction

In the CSD analysis, comparisons of the CWP group and the HC group were performed (6,239 voxels   ×   7 frequency bands). In the FC analysis, 6 FCs   ×   7 frequency bands in the DMN and 3 FCs   ×   7 frequency bands in the SN were analyzed. Therefore, to correct for multiple comparisons, the statistical nonparametric mapping (SnPM) method of eLORETA (36) was applied to identify cortical voxels that showed significant group differences in the statistical three-dimensional image. SnPM performs 5,000 permutations to determine the critical probability threshold for observed t-values, correcting for multiple comparisons across all voxels and all frequencies without relying on Gaussianity (37). eLORETA does not rely on “distribution assumptions” and provides adjusted t-critical values that are effective in controlling Type I error (38). To validate the statistical robustness of the identified neurophysiological correlations, this permutation procedure functions as a rigorous surrogate test by comparing the observed statistics against a null distribution generated from randomized data labels (39). This ensures that the reported associations are not due to random chance or spurious correlations.

In addition to the above, because the correlation analysis was performed with six self-reported scores, subsequent statistical evaluations using Spearman's rank correlation were performed using IBM SPSS Statistics (Version 27; IBM Corp., Armonk, NY, USA) to further correct for the multiplicity of these clinical measures. Based on the correlation results obtained via SnPM, CSD values were extracted from the voxels showing the maximum effect sizes within significant clusters to capture the peaks of neurophysiological relationships. In contrast, FC values were derived from an ROI-to-ROI analysis (incorporating all nearest voxels) to represent broader network dynamics. Subsequently, to assess the stability of these findings, the bootstrap method was applied; following 1,000 resampling runs, bias-corrected and accelerated confidence intervals (BCa CI) were calculated. To account for the multiplicity of the clinical scales, a Bonferroni correction was applied, resulting in a significance level of *α* < 0.0083. Furthermore, to determine whether the observed neurophysiological associations reflected the unique variance of sensory or affective pain dimensions, partial correlation analyses were performed as a sensitivity analysis, with the statistical significance level set at the standard threshold of *p* < 0.05. In these models, when examining one pain dimension (e.g., sensory), the alternate pain dimension (e.g., affective) and psychological distress scores (BDI and STAI) were included as control variables. This approach allowed for the isolation of the specific contribution of each neural correlate while accounting for potential psychological confounding factors.

### 2.6 Ethics approval

The open data used in this study were acquired with the approval of the Ethics Committee of the Faculty of Medicine, Technical University of Munich, in accordance with relevant guidelines and regulations. The study used anonymized open data, and no additional ethical review was required.

## 3 Results

### 3.1 Participant characteristics and clinical profiles

The participants’ demographic and clinical information are shown in Table 1. This study analyzed 20 participants with CWP (15 women; mean age, 51.3 ± 11.2 years) and 22 HC participants (20 women; mean age, 47.4 ± 10.8 years). Within the CWP group, correlation analysis revealed a significant positive association between the sensory and affective scores of the SF-MPQ (r = 0.671, *p* < 0.01).

**Table 1:** Participants’ demographic and clinical information.

Characteristics	CWP (*n* = 20)	HCs (*n* = 22)
Gender (men/women)	(5/15)	(2/20)
	Mean ± SD	Mean ± SD
Age (years)	51.3 ± 11.2	47.4 ± 10.8
Current pain intensity (0–10)	6.0 ± 2.1	-
SF-MPQ-sensory score	18.1 ± 5.5	-
SF-MPQ-affect score	5.8 ± 2.7	-
SF-MPQ-total score	32.9 ± 7.9	-
BDI-II score	21.5 ± 8.1	3.6 ± 4.0
STAI score	87.9 ± 16.5	63.4 ± 10.0

Data are presented as mean ± standard deviation (SD). Current pain intensity was originally recorded using a visual analogue scale ranging from 0 (no pain) to 100 (worst imaginable pain), with scores subsequently divided by 10 to derive a 0–10 numerical rating scale (NRS) for analysis. Note that pain-related measures were collected exclusively for the CWP group.

CWP, chronic widespread pain; HCs, healthy controls; SD, standard deviation; SF-MPQ, short-form McGill Pain Questionnaire; BDI-II, Beck Depression Inventory–Second Edition; STAI, State-Trait Anxiety Inventory.

### 3.2 Group comparisons of current source density and its association with self-reported scores

**Figure 1:**
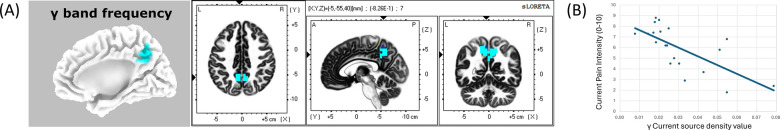
Correlation between CSD and current pain intensity in patients with CWP. **(A)** eLORETA statistical map illustrating significant negative correlations between CSD in the γ band (30.5–50.0 Hz) and current pain intensity. **(B)** Distribution map of CSD values for the voxel with the largest negative correlation between γ band activity and current pain intensity. The localized clusters are centered on the PCC/precuneus. Abbreviations: CSD, current source density; eLORETA, exact low–resolution electromagnetic tomography; NRS, numerical rating scale; PCC, posterior cingulate cortex.

There were no statistically significant differences in CSD between the CWP group and the HC group (Table 2). In the correlation analysis of each band and the pain-related scores in the CWP group, there was a significant negative correlation between the *γ* band CSD values of the PCC/precuneus and the current pain intensity (*p* < 0.001, maximum effect size across all voxels: r = −0.743) (Figure 1). The 95% BCa CI calculated using the bootstrap method in the correlation analysis between the CSD in the *γ* band and the current pain intensity was in the range of −0.903 and −0.477 (Table 3). The bias values calculated using the bootstrapping method were 0.027, and the SE values were 0.106. Additionally, the *β*2 band showed a nominally significant correlation (*p* = 0.016, maximum effect size across all voxels: r = −0.532); however, this did not survive the Bonferroni correction (Supplementary Figure S1). The 95% BCa CI calculated using the bootstrap method in the correlation analysis between the CSD in the *β*2 band and the current pain intensity was in the range of −0.848 and −0.046 (Table 3). The bias values calculated using the bootstrapping method were 0.016, and the standard error (SE) values were 0.212. To allow for an assessment of the overall pattern of effects beyond the strictly thresholded significant voxels, the anatomical distribution of the correlation for both *γ* and *β* 2 bands at a relaxed significance threshold (*p* < 0.10, uncorrected) is further illustrated in Supplementary Figure S2. Furthermore, there were no statistically significant correlations between CSD values in any frequency band and psychological assessment scores, including depression (BDI) and anxiety (STAI). To further evaluate the unique contribution of pain intensity to this finding, a partial correlation analysis was performed. The association between *γ* band CSD in the PCC/precuneus and current pain intensity remained significant even after controlling for the SF-MPQ affective score, BDI, and STAI (r = −0.641, *p* = 0.006).

**Table 2:** Summary of independent t-tests for whole-brain CSD comparison between CWP and HC groups.

Analysis method	Statistical parameter	Value	Peak localization, BA (X, Y, Z)
Permutation-corrected statistics (global)	Extreme *p*-value (Two-tailed, permutation-corrected)	*p* = 0.721	-
	Critical t-threshold for significance (*p* < 0.05)	t = ±3.684	-
Peak global trend (Uncorrected)	Maximal absolute *t*-statistic	t = 2.312	-
	Estimated peak effect size (Cohen's d)	d ≈ 0.37	-
Peak t-statistics per frequency band (Uncorrected t-statistics)	*δ* (1.5–4.0 Hz)	t = −1.523	Anterior Cingulate,BA 25 (5, 20, −5)
	*θ* (4.5–7.5 Hz)	t = −2.159	Anterior Cingulate, BA 33 (5, 10, −25)
	*α*1 (8.0–10.0 Hz)	t = −2.312	Middle Frontal Gyrus, BA9 (35, 15, 35)
	α2 (10.5–13.0 Hz)	t = 1.855	Middle Temporal Gyrus, BA 21 (65, −50, −5)
	*β*1 (13.5–20.0 Hz)	t = −1.462	Anterior Cingulate, BA 25 (0, 0, −5)
	β2 (20.5–30.0 Hz)	t = −1.367	Subcallosal Gyrus, BA 25 (0, 5, −15)
	*γ* (30.5–50.0 Hz)	t = 1.939	Superior Frontal Gyrus, BA 6 (−20, 15, 65)

Statistical significance was evaluated using eLORETA's non-parametric randomization (permutation) test with 5,000 permutations, providing a stringent global correction for multiple comparisons across all brain voxels and all frequency bands (delta, theta, alpha 1, alpha 2, beta 1, beta 2, and gamma) simultaneously. The band-specific peak trends represent the maximal absolute t-statistics observed within each frequency band before global correction, where negative and positive values denote directional trends of CWP < HC and CWP > HC, respectively. The global peak t-statistic across all analyzed voxels and frequency bands was observed in the alpha 1 band (t = 2.312). Cohen's d was estimated based on the maximal absolute t-statistic and the total sample size (*n* = 42).

CSD, current source density; CWP, chronic widespread pain; HC, healthy controls; BA, Brodmann Area.

**Table 3:** Summary of correlation analyses between neurophysiological indices (CSD and FC) and clinical pain scores in the CWP group (*n* = 20).

Analysis	Frequency band	Brain region/ connection	MNI coordinates (X, Y, Z)	r-value	BCa CI		*p*-value
					Lower limit	Upper limit	
CSD	β2	PCC/Precuneus	(10, −70, 35) Maximum effect size voxel	−0.532	−0.848	−0.046	*p* = 0.016
	γ	PCC/Precuneus	(−5, −55, 40) Maximum effect size voxel	−0.743	−0.903	−0.477	*p* < 0.001*
FC	δ	ACC – rAI	(5, 30, 25) – (30, 20, −5) All nearest voxels	0.647	0.282	0.878	*p* = 0.002*

Results are presented for correlation analyses conducted between EEG-derived parameters and clinical pain assessments. All brain locations are defined by MNI coordinates. For CSD, values were extracted from the maximum effect size voxel at the peak coordinate. For FC, values were calculated based on all nearest voxels associated with the designated ROI center. For current source density (CSD), the Montreal Neurological Institute (MNI) coordinates (X, Y, Z) correspond to the peak voxel within the cluster showing the strongest correlation. The r-values represent Spearman's rank correlation coefficients. Findings that reached nominal significance (*p* < 0.05) but did not survive the Bonferroni correction are also included for transparency. The 95% BCa CI was calculated using a bootstrapping method with 5,000 iterations.

BCaCI, bias-corrected and accelerated confidence interval; CSD, current source density; CWP, chronic widespread pain; FC, functional connectivity; MNI, Montreal Neurological Institute; SnPM, statistical nonparametric mapping.*p < 0.01.

### 3.3 Group comparisons of functional connectivity and its association with self-reported scores

**Figure 2:**
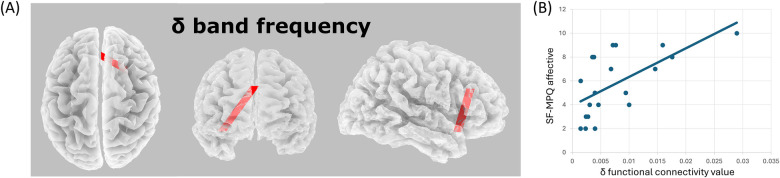
Correlation between δ band FC (ACC-rAI) and SF-MPQ affective item score. **(A)** The red line represents the linear regression fit to the data, demonstrating a positive relationship between increased ACC-rAI δ band connectivity and higher scores on the SF-MPQ emotional expression item. Each data point represents a single participant with CWP. **(B)** Distribution map illustrating the significant positive correlation between FC in the δ band (1.5-4.0 Hz) between the ACC and rAI and the score on the affective item of the SF-MPQ. Abbreviations: FC, functional connectivity; ACC, anterior cingulate cortex; rAI, right anterior insula; SF-MPQ, short-form McGill Pain Questionnaire; CWP, chronic widespread pain.

There were no statistically significant differences in the FC of the DMN and SN between the CWP group and the HC group (Table 4). In the correlation analysis of the CWP group, there was a significant positive correlation between the FC value of the *δ* band of the ACC–rAI and the score on the affective representation item of the SF-MPQ (*p* = 0.002, effect size: r = 0.647) (Figure 2). The 95% BCa CI calculated using the bootstrap method in the correlation analysis between FC and the score on the SF-MPQ affective representation item was 0.282–0.878 (Table 3). The bias values calculated using the bootstrapping method were −0.017, and the SE values were 0.153. Similar to the CSD results, no significant correlations were observed between the FC values of the DMN or SN and the BDI or STAI scores. Furthermore, a partial correlation analysis confirmed that the association between the *δ* band FC of the ACC–rAI and the SF-MPQ affective score remained significant after controlling for SF-MPQ sensory score, BDI, and STAI (r = 0.507, *p* = 0.038).

**Table 4:** Summary of independent t-tests for DMN and SN FC comparison between CWP and HC groups.

Analysis method	Statistical parameter	Value (DMN)	Value (SN)
Permutation-corrected statistics (Global)	Extreme *p*-value (Two-tailed, permutation-corrected)	*p* = 0.991	*p* = 0.240
	Critical t-threshold for significance (*p* < 0.05)	t = ±3.229	t = ±3.005
Peak global trend (Uncorrected)	Maximal absolute *t*-statistic	t = 1.509	t = 2.409
	Estimated peak effect size (Cohen's d)	d ≈ 0.24	d ≈ 0.38
Peak t-statistics per frequency band (Uncorrected t-statistics)	δ (1.5–4.0 Hz)	t = −1.146 [1] – [2]	t = −1.829 [1] – [2]
	θ (4.5–7.5 Hz)	t = −1.375 [1] – [4]	t = −1.586 [1] – [3]
	α1 (8.0–10.0 Hz)	t = −1.413 [1] – [4]	t = 0.366 [1] – [2]
	α2 (10.5–13.0 Hz)	t = 1.280 [1] – [4]	t = 2.409 [1] – [2]
	β1 (13.5–20.0 Hz)	t = −1.309 [3] – [4]	t = −1.338 [1] – [3]
	β2 (20.5–30.0 Hz)	t = 0.976 [2] – [4]	t = −0.900 [1] – [2]
	γ (30.5–50.0 Hz)	t = −1.509 [3] – [4]	t = 1.900 [2] – [3]

Statistical significance was evaluated using eLORETA's non-parametric randomization (permutation) test with 5,000 permutations, providing a correction for multiple comparisons across all analyzed functional connectivity pairs (6 within the DMN and 3 within the SN) and all frequency bands (*δ, θ, α* 1, *α* 2, *β* 1, *β* 2, and *γ*) simultaneously. The band-specific peak trends represent the maximal absolute t-statistics observed within each frequency band before global correction, where positive and negative values denote directional trends of CWP > HC and CWP < HC, respectively. To ensure visual clarity, anatomical regions are represented by numerical indices, and functional connections are denoted by the corresponding pairs of these indices. (DMN: MedFrGyrus[1], PostCingGyrus[2], LeftInfParLobe[3], RightInfParLobe[4], SN: aCC[1], laINS[2], raINS[3]).

FC, functional connectivity; CWP, chronic widespread pain; HC, healthy controls; DMN, default mode network; SN, salience network.

## 4 Discussion

This exploratory study investigated the neurophysiological components of CWP by examining CSD and FC in resting-state EEG data, comparing patients with CWP and HCs. Contrary to certain expectations based on the fMRI literature, no statistically significant differences in CSD or FC were found when comparing the CWP group and the HC group as a whole. This discrepancy likely results from the high clinical heterogeneity of CWP, which may have led to a “wash-out” effect in group-level mean comparisons. This conceptual explanation is supported by our band-specific group-level results (Tables 2, 4), which revealed varying and non-significant directional effects across frequency bands. Given the exploratory nature of this study, our whole-brain voxel-wise approach was intended for hypothesis generation rather than definitive biomarker validation. For instance, while no results survived global permutation correction, the SN showed a peak trend toward increased connectivity (t = 2.409, d ≈ 0.38) even in the absence of categorical significance. Such variance indicates that individual differences may have limited the detection of a consistent neurophysiological pattern at the group level, creating a substantial inferential burden that could hinder the identification of consistent findings. Indeed, a recent systematic review has highlighted that resting-state EEG findings across chronic neuropathic pain studies often show no consistent trend in group-level biomarkers or correlations with pain intensity, largely due to high inter-study heterogeneity and the influence of confounding factors (40). Furthermore, these EEG correlates may be more sensitive to the fluctuating “state” of individual pain intensity at the time of recording rather than representing a stable “trait” of the diagnostic category. Consequently, our findings must be interpreted as neurophysiological correlates of individual pain experience within the patient group, rather than as disease-specific mechanisms of CWP as a diagnostic entity.

The significant negative correlation observed between the CSD values in the *γ* bands (and nominally in the *β*2 band) of the precuneus/PCC and current pain intensity is noteworthy. The PCC, a major node of the DMN, is involved in self-referential thinking and pain processing (41). Past studies have shown changes in the activity and connectivity of the DMN in chronic pain conditions, including fibromyalgia (5, 42). In addition, a previous study showed that pain sensitivity and gray matter density in the PCC, precuneus, intraparietal sulcus, and inferior parietal lobule were negatively correlated (43). While group-level comparisons did not yield significant differences (Table 2), possibly due to the aforementioned clinical heterogeneity, the negative correlation observed in this study suggests that decreased activity peaking in the PCC/precuneus at rest may be associated with an increase in current pain intensity in patients with CWP. Importantly, our partial correlation analysis demonstrated that this association remained significant even after controlling for affective pain scores and psychological distress, underscoring its robust relationship with the sensory dimension of pain. Given the spatial resolution of our methodology, this finding likely reflects an association involving the posterior midline hubs of the DMN rather than a uniquely localized anatomical effect. While the functional significance remains to be elucidated, it is possible that decreased baseline activity in the PCC may reflect neural processes potentially related to the brain's attempt to modulate or suppress ongoing pain signals. It may also indicate that in people with high levels of pain, the DMN is less involved in typical functions at rest, possibly because of the dominance of pain-related processing. We propose that in the context, the PCC may exhibit a functional shift from its typical self-referential functions to processes potentially related to the persistent pain burden. Given the exploratory nature of this study, this finding—while most prominent in the PCC/precuneus—likely represents a broader neurophysiological correlate of individual sensory pain state within the posterior DMN hubs, rather than a uniquely localized or categorical biomarker for the disorder.

The positive correlation in this study between FC in the *δ* band between the ACC and the rAI and the score on the affective representation item of the SF-MPQ is also interesting. The ACC and insula are key components of the SN, which is involved in the detection of and response to salient stimuli (44). The importance of functional connectivity within these pain-related hubs has been highlighted in recent studies on fibromyalgia (45). In our group-level comparison of the SN (Table 4), we observed a peak t-statistic of 2.409 (d ≈ 0.38), which represents a medium effect size within this specific sample; however, the lack of global significance reinforces the idea that these relationships are more robustly captured through individual pain scores than through categorical group classifications. Increased connectivity between these regions in the *δ* band may indicate enhanced communication related to the affective aspects of pain. The affective representation item of the SF-MPQ captures the affective aspects of pain, such as distress, anxiety, and fear. Therefore, the results of this study suggest that in patients with CWP, stronger connectivity within the SN, especially in the *δ* band, is associated with an increased affective burden of pain. Crucially, this association remained significant in our partial correlation analysis even after accounting for current pain intensity and psychological distress (BDI and STAI), supporting the view that this network specifically relates to the affective-motivational dimension of the pain experience. However, given the correlational nature of this exploratory study, no causal relationship can be inferred. This interpretation is consistent with the understanding that chronic pain is not simply a sensory experience but is intertwined with affective and cognitive processes (34). As with the CSD findings, this salience network connectivity (r = 0.647) suggests that these hubs could potentially shift their functional roles toward processing the affective dimension of pain. Nevertheless, given the shared variance between sensory and affective pain dimensions, these results should be interpreted as preliminary neurophysiological patterns rather than as evidence for a strictly dissociated mechanism.

Several limitations need to be considered when interpreting the results of this study. First, CWP is a disease with diverse symptoms, and the neurophysiological mechanisms may vary greatly from individual to individual. While the variance in our band-specific group trends (Tables 2, 4) provides analytical support for this heterogeneity, our current sample size precluded more direct analytical subgrouping. This diversity can obscure group-level differences in neurophysiological measurements through the aforementioned “wash-out” effect. In the future, subgrouping patients on the basis of pain phenotype, psychological profile, or other clinical characteristics may reveal clearer patterns. Second, the sample size (20 patients with CWP and 22 HC participants) was relatively small, which may have limited the statistical power to detect subtle group differences and increased the risk of Type II errors. This sample size resulted from our strict selection of a unified subset within the Technical University of Munich database to ensure clinical homogeneity. While this focused approach minimizes the confounding effects of clinical heterogeneity and enhances the internal validity of our exploratory findings, larger-scale studies are required to confirm these results. Third, the use of 19 EEG channels for source estimation, while clinically practical, provides lower spatial resolution compared to high-density EEG systems. This limitation may affect the precision of anatomical localization for the observed spectral changes (46). Furthermore, given the exploratory nature of this whole-brain strategy, it remains unclear whether the observed associations are uniquely localized to the PCC or the SN, or whether they reflect more global spatial patterns; thus, caution is warranted when interpreting regional specificity based on these data. Although eLORETA minimizes localization error, future studies utilizing high-density EEG are required to validate these findings with greater spatial precision. Fourth, this study relied on eyes-closed resting-state EEG, which may not fully capture the dynamic neural processes underlying CWP. Future studies may benefit from incorporating pain-induced EEG paradigms or analyzing EEG data during specific tasks. Fifth, while we examined the potential influence of depression and anxiety using the BDI and STAI, and employed partial correlation analyses to control for these scores, it should be noted that these assessments may not capture the full complexity of psychological influences. Consequently, the possibility remains that unmeasured psychological or cognitive factors could still influence the results. Given their known influence on PCC and salience network activity, the interplay between psychological distress and pain neurophysiology remains a critical consideration. Furthermore, while the SF-MPQ was used to differentiate between the sensory and affective dimensions of pain, these measures are inherently correlated. Although our partial correlation analyses were used to isolate the unique variance of each dimension, their complete independence is difficult to achieve in a clinical population, and larger studies are needed to further validate this dissociation. Sixth, the exploratory strategy across multiple frequency bands in a small sample creates a substantial inferential burden, necessitating a cautious interpretation of the observed associations. Seventh, the dataset did not include specific records of medication use. Since various pharmacological agents commonly prescribed for chronic pain, such as antidepressants or anticonvulsants, are known to alter EEG power spectra and functional connectivity, the inability to control for these effects is a notable limitation. This factor should be taken into account when interpreting the observed neurophysiological patterns. Finally, because this study has a cross-sectional design, our ability to make inferences about causality is limited.

In conclusion, this study provides exploratory evidence of potential associations between specific EEG correlations and subjective pain experience in patients with CWP. As no significant group differences were found, these findings represent exploratory electrophysiological correlates of individual pain burden rather than distinct biomarkers. Although no significant group differences in CSD or FC were found between patients with CWP and HCs, correlation analysis revealed that decreased CSD in the PCC and precuneus (primarily in the *γ* band, with a similar trend in the *β*2 band) was associated with current pain intensity, and increased FC between ACC and rAI (*δ* band) was associated with the affective aspects of pain. These findings suggest that neurophysiological correlations obtained from EEG may provide insights into individual differences in pain experience in CWP. Future studies should include larger, more homogeneous patient samples, and longitudinal studies are needed to determine whether these EEG correlations of pain state can predict treatment response or disease progression.

## Data Availability

Publicly available datasets were analyzed in this study. This data can be found here: May ES, Gil Ávila C, Ta Dinh S, Heitmann H, Hohn VD, Nickel MM, et al. Dynamics of brain function in patients with chronic pain assessed by microstate analysis of resting-state electroencephalography. Pain. 2021;162(12):2894-908.
