# Supplementary material for: Exploratory EEG correlates of sensory and affective pain dimensions in patients with chronic widespread pain
Source: Front Pain Res (Lausanne). 2026 Jul 16;7:1705011. doi: 10.3389/fpain.2026.1705011 (PMC13422479; doi:10.3389/fpain.2026.1705011)
Supplement: Supplementary file 1 [file Datasheet1.pdf]

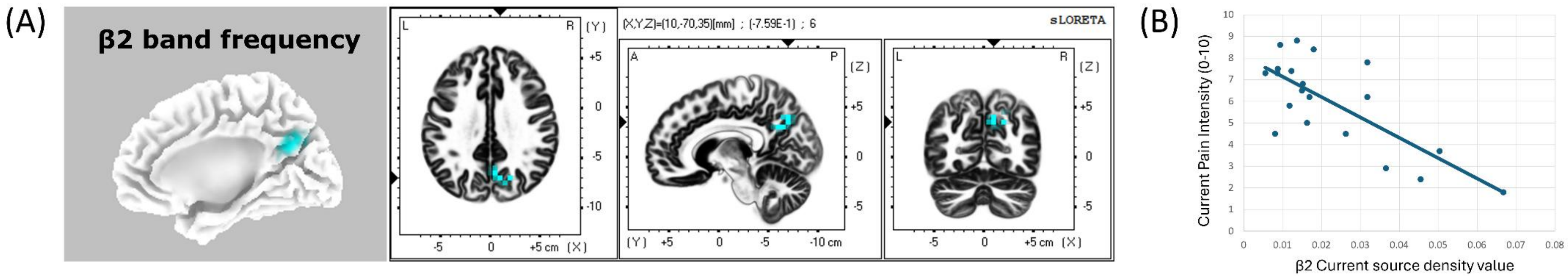

Figure S1. Nominal correlation between β2 band CSD and current pain intensity.

(A) eLORETA statistical map showing a nominal negative correlation between CSD in the β2 band (20.5–30.0 Hz) and current pain intensity in the PCC/precuneus ($p < 0.05$, uncorrected). (B) Scatter plot illustrating the relationship at the peak voxel. While this association reached nominal significance, it did not survive the Bonferroni correction ($p > 0.0083$) applied in our primary analysis. Each data point represents an individual participant with CWP. Abbreviations: CSD, current source density; eLORETA, exact low-resolution electromagnetic tomography; PCC, posterior cingulate cortex; CWP, chronic widespread pain.

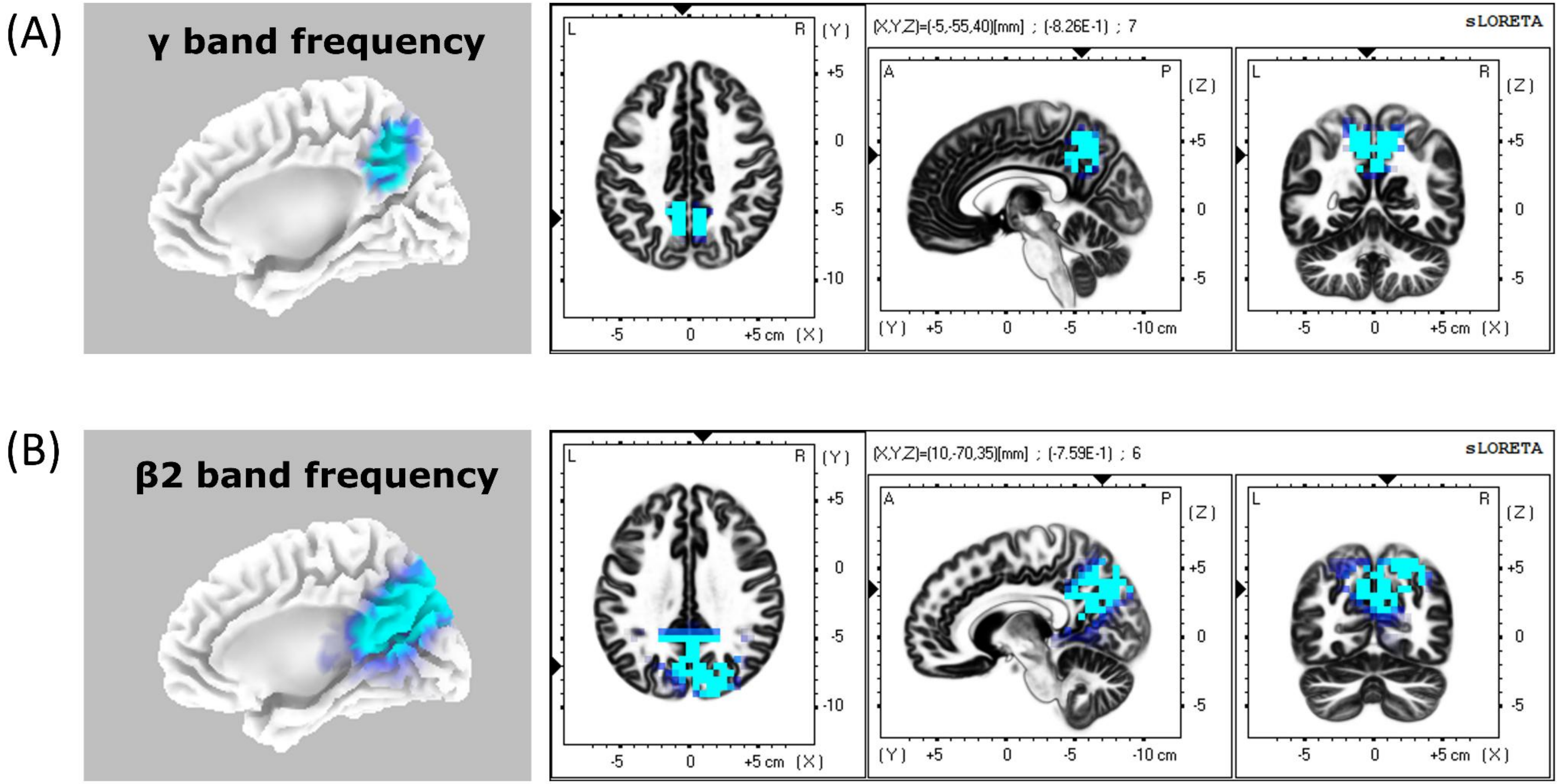

Figure S2. Spatial extent of γ band CSD correlation at a relaxed threshold.

This map illustrates the anatomical distribution of the negative correlation between γ band CSD (30.5–50.0 Hz) and current pain intensity at a relaxed significance threshold ($p < 0.10$, uncorrected). (B) The anatomical distribution for the β2 band CSD (20.5–30.0 Hz) is also shown at the same threshold. These visualization demonstrates a broader spatial extent of neurophysiological correlations in the PCC/ precuneus and its surrounding regions.

Abbreviations: CSD, current source density; PCC, posterior cingulate cortex.
